# Genome-wide shRNA screening identifies host factors involved in early endocytic events for HIV-1-induced CD4 down-regulation

**DOI:** 10.1186/s12977-014-0118-4

**Published:** 2014-12-13

**Authors:** Alessia Landi, Jolien Vermeire, Veronica Iannucci, Hanne Vanderstraeten, Evelien Naessens, Mostafa Bentahir, Bruno Verhasselt

**Affiliations:** Department of Clinical Chemistry, Microbiology, and Immunology, Ghent University and Ghent University Hospital, De Pintelaan 185, B-9000 Gent, Belgium; Present address: Department of Microbiology, Icahn School of Medicine at Mount Sinai, New York, NY USA; Present address: Centre de Technologies Moléculaires Appliquées, Ecole de Santé Publique, Brussels, Belgium

**Keywords:** HIV-1, shRNA, CD4, Nef, Endocytosis

## Abstract

**Background:**

Down-modulation of the CD4 receptor is one of the hallmarks of HIV-1 infection and it is believed to confer a selective replicative advantage to the virus *in vivo*. This process is mainly mediated by three viral proteins: Env, Vpu and Nef. To date, the mechanisms that lead to CD4 depletion from the surface of infected cells during HIV-1 infection are still only partially characterized. In this study, we sought to identify and characterize cellular host factors in HIV-1-induced CD4 down-modulation.

**Results:**

To identify host factors involved in CD4 down-regulation, we used a whole genome-targeting shRNA lentiviral library in HeLa CD4+ cells expressing Nef as an inducer of CD4 down-modulation. We identified 55 genes, mainly encoding for proteins involved in various steps of clathrin-mediated endocytosis. For confirmation and further selection of the hits we performed several rounds of validation, using individual shRNA lentiviral vectors with a different target sequence for gene knock-down in HIV-1-infected T cells. By this stringent validation set-up, we could demonstrate that the knock-down of DNM3 (dynamin 3), SNX22 (sorting nexin 22), ATP6AP1 (ATPase, H+ Transporting, Lysosomal Accessory Protein 1), HRBL (HIV-Rev binding protein Like), IDH3G (Isocitrate dehydrogenase), HSP90B1 (Heat shock protein 90 kDa beta member 1) and EPS15 (Epidermal Growth Factor Receptor Pathway Substrate 15) significantly increases CD4 levels in HIV-infected SupT1 T cells compared to the non-targeting shRNA control. Moreover, EPS15, DNM3, IDH3G and ATP6AP1 knock-down significantly decreases HIV-1 replication in T cells.

**Conclusions:**

We identified seven genes as cellular co-factors for HIV-1-mediated CD4 down-regulation in T cells. The knock-down of four out of seven of these genes also significantly reduces HIV-1 replication in T cells. Next to a role in HIV-mediated CD4 down-regulation, these genes might however affect HIV-1 replication in another way. Our findings give insights in the HIV-1-mediated CD4 down-regulation at the level of the plasma membrane and early endosomes and identify four possible new HIV-1 replication co-factors.

**Electronic supplementary material:**

The online version of this article (doi:10.1186/s12977-014-0118-4) contains supplementary material, which is available to authorized users.

## Background

The integral membrane protein CD4 is expressed in hematopoietic cells, primarily on the surface of T-cell progenitors, T helper cells and cells belonging to the monocyte/macrophage lineage. In T lymphocytes this protein acts as a co-receptor with the T-cell receptor (TCR) for recognition of class II major histocompatibility complex associated with antigenic peptide, participating in T-cell development and activation. Following T-cell activation, CD4 is down-modulated via clathrin-mediated endocytosis and is then sorted from early endosomes into late endocytic organelles where it is degraded [1].

CD4 also serves as the primary receptor for HIV infection and CD4 down-regulation is an important determinant of viral replication, pathogenesis and disease progression [2-4]. HIV-mediated CD4 down-modulation can therefore be considered as an interesting therapeutic target. Three HIV-1 proteins are known to play a role in CD4 down-modulation during HIV-1 infection: Env, Vpu and Nef. The development of multiple mechanisms for CD4 down-regulation by HIV points to the importance of this phenomenon. Each of these proteins can reduce CD4 surface expression independently and their concerted action is able to eliminate CD4 expression on the surface of infected T cells almost completely [5,6]. The effect of Env on CD4 expression is mainly exerted by the immature viral envelope complex gp160, which retains the nascent CD4 molecules in the endoplasmic reticulum (ER) [5,7]. The effects of Nef and Vpu are quantitatively and qualitatively distinct. Nef acts on CD4 expression early in the viral life cycle, enhancing CD4 internalization from the cell surface by linking it to the AP-2 clathrin adaptor [8,9]. Via its interaction with β-COP, Nef appears to target the internalized receptor to endosomes [10] and subsequently to the Multivesicular Body Pathway (MVB) in an Endosomal Sorting Complex Required for Transport (ESCRT)-dependent manner [11]. Vpu is expressed later in the life cycle of the HIV-1 virus and causes the degradation of newly synthesized CD4 receptor, retained in the ER by the envelope glycoprotein gp160. It has been shown that the Vpu-mediated degradation of CD4 happens via two independent mechanisms: the viral protein can either trigger proteasomal degradation of the receptor by linking it to β-TrCP, a component of the SCF^β-TrCP^ E3 Ub ligase complex [12]. Vpu can also act in a variant Endoplasmic Reticulum-Associated Degradation (ERAD) pathway to independently mediate the retention of CD4 in the ER, a function traditionally attributed to Env [13-15]. However, the exact pathways used by the HIV-1 proteins to divert the internalized CD4 molecules to lysosomal degradation and hamper their recycling remain to be identified.

Genome-wide RNA interference screenings are a useful tool to discover the role of proteins in a given cellular process. Several approaches have been described in which libraries of short interfering RNAs (siRNAs) and short hairpin RNAs (shRNAs) were used to identify new cellular factors involved in HIV-1 infection and replication [16]. We chose to use a genome-wide shRNA screening to identify new cellular factors involved in CD4 down-modulation, using the expression of HIV-1 Nef as an inducer of a rapid and efficient down-regulation of CD4 from the cell surface in CD4-positive HeLa cells (HeLa CD4++). In cells expressing a shRNA sequence which blocks the expression of a gene involved in CD4 down-modulation, we expect high CD4 cell surface staining despite the expression of Nef. Positive hits selected in this screening step were subsequently validated in a HIV-1-infected T cell line to evaluate their role in HIV-1-mediated CD4 down-modulation in a more relevant model. To exclude off-target effects as much as possible, validation of each positive hit was carried out using different shRNA targeting sequences compared to the ones used in the screening. We identified seven genes for which knock-down significantly rescued CD4 expression in HIV-1 infected SupT1 cells. These genes are: DNM3 (dynamin 3), SNX22 (sorting nexin 22), ATP6AP1 (ATPase, H+ Transporting, Lysosomal Accessory Protein 1), HRBL (HIV-Rev binding protein Like), IDH3G (Isocitrate dehydrogenase), HSP90B1 (Heat shock protein 90 kDa beta member 1), EPS15 (Epidermal Growth Factor Receptor Pathway Substrate 15). Four genes (DNM3, EPS15, ATP6AP1, IDH3G) out of seven were also found to significantly reduce HIV-1 replication upon knock-down.

## Results

### Genome-wide shRNA screening

**Figure 1:**
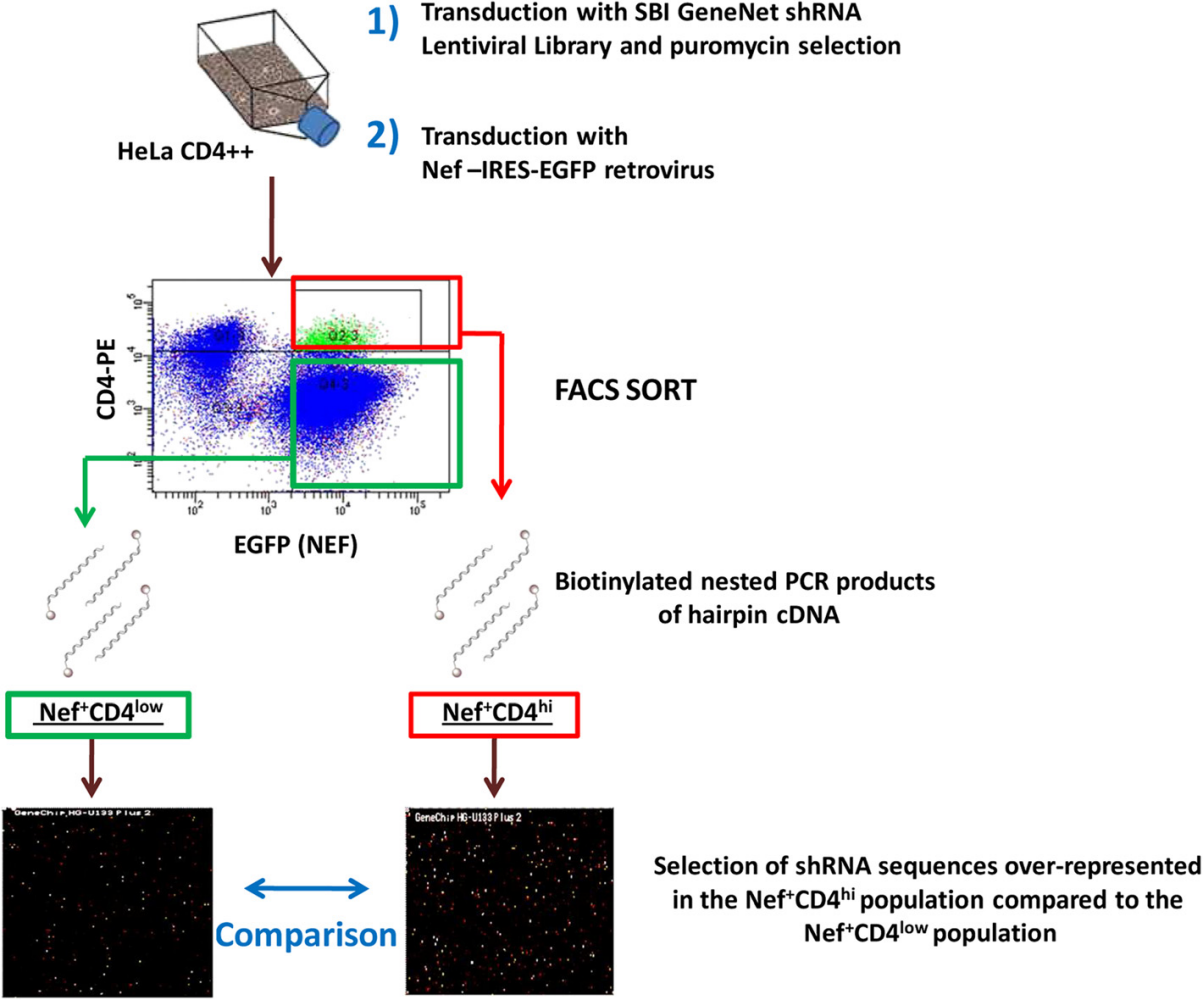
**Schematic representation of the genome-wide shRNA screening workflow.** HeLa CD4++ cells were transduced with a pooled shRNA lentiviral library. Productively transduced cells were selected with puromycin and transduced with a retroviral vector for the expression of HIV-1 Nef and eGFP as a reporter gene. Cells that maintained high CD4 surface levels despite the expression of Nef (Nef(eGFP)^+^CD4^hi^, red box) and the Nef(eGFP)^+^CD4^low^ population (green box) were sorted by FACS. The shRNA hairpins present in the cDNA obtained from the two sorted populations were amplified by nested PCR with biotinylated primers. The samples obtained were hybridized on different Affymetrix U133 v2-plus microarrays and the results compared. shRNA sequences enriched in the Nef(eGFP)^+^CD4^hi^ cells were selected and further analyzed with pathway analysis tools to obtain a final list of 55 genes to be further validated together with 20 genes selected from literature.

We performed a shRNA genome-wide screening to discover cellular factors involved in CD4 down-regulation in Nef-expressing cells. CD4 positive HeLa cells were transduced with a pooled commercial lentiviral vector library (System Biosciences) encoding shRNAs targeting the whole genome under a puromycin resistance selection marker. The library contained 200,000 shRNA sequences targeting around 47,000 human transcripts. Viral titer was adjusted to a multiplicity of infection of 0.3. In this way efficiently transduced cells, selected by puromycin resistance, were expected to express only one shRNA sequence. These selected cells were subsequently transduced with a retrovirus encoding Nef and eGFP as a reporter gene [17]. The expression of Nef decreases cell surface CD4 levels, but not in the few cells that express a shRNA blocking the expression of a gene important for CD4 down-regulation. Therefore, eGFP positive cells (expressing Nef) with high surface CD4 levels (Nef(eGFP)^+^CD4^hi^) were sorted with flow cytometry, as well as the population of cells expressing Nef and expressing low CD4 levels (Nef(eGFP)^+^CD4^low^). The expressed shRNA sequences were amplified with biotinylated primers from the cDNA of these two populations, and PCR products were hybridized to a microarray compatible with the lentiviral library. The shRNA sequences that were over-represented in Nef(eGFP)^+^CD4^hi^ cells by at least two-fold compared to the Nef(eGFP)^+^CD4^low^ population were selected using criteria as the magnitude of the ratio, the reproducibility in 4 independent genome-wide screenings, and their biological function (Figure 1). Among the gene products targeted by the shRNA sequences over-represented in the Nef^+^CD4^hi^ cells, several known Nef co-factors like AP2A2 (Adaptor protein 2, subunit alpha), clathrin chains CLTB (Clathrin, light chain) and CLTC1 (Clathrin, heavy chain like 1) were found, which can be considered as a proof of validity of the screening approach. We obtained a shortlist of 55 genes, most of them involved in different stages of the endocytic, recycling, Trans Golgi Network (TGN) and lysosomal degradation pathways. To this, 20 genes (mainly other members of the families of the genes obtained from the screenings) or additional controls, were added (see Additional file 1). The final set of 75 genes was used in the further selection, validation and confirmation steps.

### Biological validation of the hits

**Figure 2:**
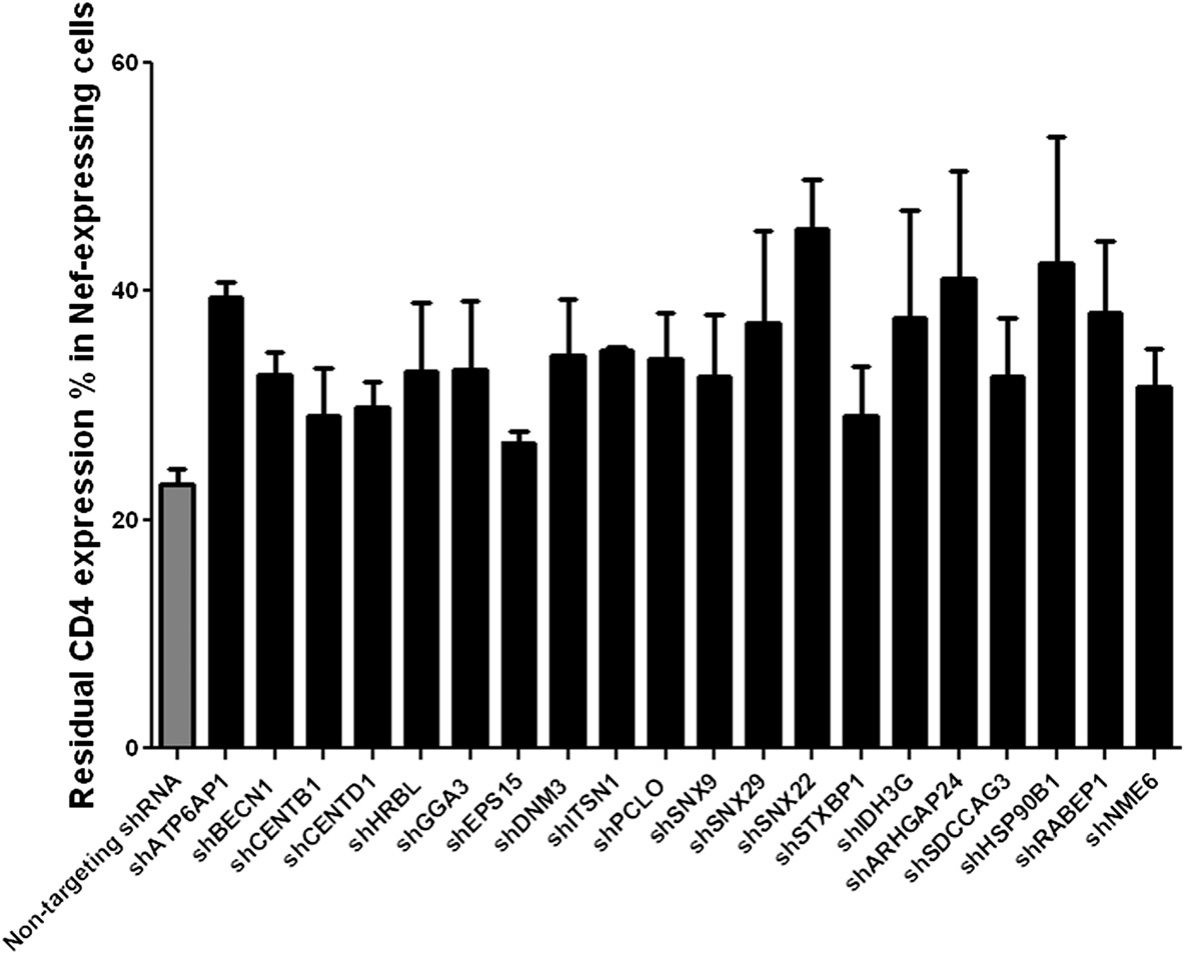
**Results of validation 1: CD4 residual expression in HeLa CD4++ expressing Nef and the shRNAs targeting the selected 20 genes.** Bar graph shows the residual CD4 expression in HeLa CD4++ expressing the shRNAs for the knock-down of the 20 selected hits and the Nef retroviral construct. The values are representative of two independent confirmation experiments and for the shRNA clone with most activity among the ones tested. The gray bar indicates the value obtained in cells transduced with the non-targeting scrambled shRNA control.

**Figure 3:**
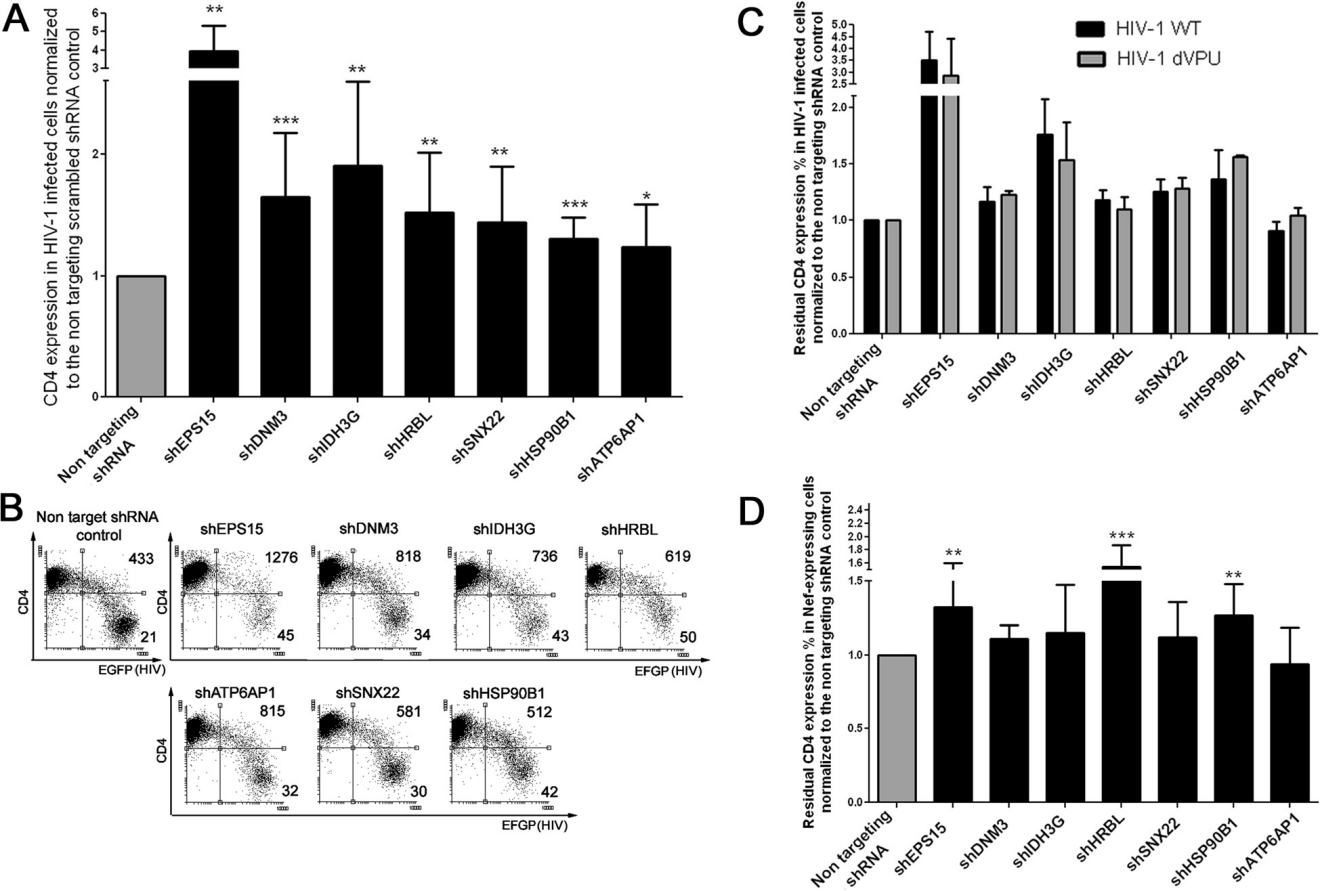
**Effect of the knock-down of the selected genes on CD4 expression in HIV-1-infected SupT1 cells. A**. The bar graph shows the residual CD4 surface expression levels in HIV-1-infected (eGFP+) SupT1 cells (calculated as described in Methods) normalized to the non-targeting shRNA scrambled control. Error bars represent standard deviation between 7 to 17 independent experiments. The values are representative of the shRNA clone with most activity among the ones tested. P values: ***p ≤ 0.0004; **p ≤ 0.0099; *p ≤ 0.038. **B**. Flow cytometry dot plots showing the CD4 surface levels in HIV-1-infected cells in a representative experiment. eGFP is the reporter for HIV-1 infection, while CD4 levels are measured by surface staining with an anti-CD4 monoclonal antibody conjugated to APC. The numbers on the FACS plots indicate the mean fluorescence intensity of CD4 gated on the upper right and lower right quadrants. **C**. The bar graph shows the residual CD4 surface expression levels in shRNA expressing SupT1 cells normalized to the non-targeting shRNA scrambled controls (calculated as described in Methods), infected with the wild type (black bars, indicated as HIV-1 WT) and the dVpu HIV-1 NL4-3 IRES-EGFP (grey bars, indicated as HIV-1 dVpu). Error bars represent standard deviation between 3 independent experiments. **D**. The bar graph shows the residual CD4 surface expression levels (calculated as described in Methods) in SupT1 cells transduced by a retroviral vector to express Nef, normalized to the non-targeting shRNA scrambled control. Error bars represent standard deviation between 7 to 16 independent experiments. P values: ***p ≤ 0.0006 ; **p ≤ 0.0011.

To confirm the biological relevance of the selected gene candidates, we used an independent validation strategy using different shRNA sequences (Mission RNAi consortium) to target the genes identified during the screening. For each of the selected genes, we individually evaluated 2 to 5 lentiviral clones, each expressing different shRNA sequences to target the gene transcript (depending on the validation status and availability by the supplier). The validation set-up involved two different approaches: the first approach (validation 1) was a confirmation of the hits in HeLa CD4++ using Nef as an inducer of CD4 down-modulation. Screening hits confirmed in HeLa cells were subsequently investigated in a more relevant system (validation 2): SupT1 T cells infected with HIV-1 engineered to express an eGFP reporter gene (Table 1). Our final interest is primarily in host proteins that are important for CD4 trafficking in HIV-1-infected cells, but in epithelial cells such as HeLa cells, CD4 trafficking might differ in some aspects from that in T cells, the main targets of HIV-1 [18]. In validation 1, we confirmed that knock-down of 20 genes out of 75 selected hits affected CD4 down-regulation (see Figure 2). These genes were further evaluated in validation 2. These 20 selected genes are briefly described in Additional file 2. In validation 2, cell surface expression of CD4 was analyzed two, four and seven days after HIV infection of SupT1 cells expressing shRNA targeting one of these 20 genes. For this, we used an HIV NL4-3 construct expressing all the HIV genes and a GFP marker gene expressed in frame with Nef, separated through an IRES sequence. The residual CD4 expression in the HIV-infected fraction (eGFP^+^) compared to uninfected cells in the same culture (eGFP^−^) was calculated as described in Methods. Seven genes were found to significantly contribute to CD4 down-regulation in HIV-infected SupT1 (Figure 3A). These genes are: DNM3 (dynamin 3; 1.7 fold increase of CD4 residual expression compared to non-targeting shRNA-expressing cells), SNX22 (sorting nexin 22; 1.5 fold), HRBL (HIV-Rev binding protein Like; 1.6 fold), IDH3G (Isocitrate dehydrogenase; 1.9 fold), HSP90B1 (Heat shock protein 90 kDa beta member 1; 1.3 fold), EPS15 (Epidermal Growth Factor Receptor Pathway Substrate 15; 4 fold) and ATP6AP1 (ATPase, H+ Transporting, Lysosomal Accessory Protein 1; 1.25 fold). The knock-down efficiencies at mRNA level were determined via qPCR for the selected shRNA clones (see Additional file 3). To investigate whether the effects observed were Nef-dependent we performed two additional sets of experiments, using different viruses and vectors. In the first, we compared CD4 down-modulation, either induced by wild type reporter virus (HIV-1 NL4-3 IRES-EGFP) or by a *vpu* -deleted derivative virus (HIV-1 NL4-3 IRES-EGFP, dVpu) in SupT1 cells knocked-down for the target genes. The residual CD4 expression in the HIV-infected cells was calculated as described above. The fold increase of CD4-expression in cells knocked down for the target genes relative to the control non-targeting shRNA-expressing cells was comparable between WT and dVpu viruses (Figure 3C), suggesting little contribution of these factors to Vpu-mediated CD4 down-regulation.

**Table 1:** **Experimental setup overview for the validation of the hits obtained from the genome-wide shRNA screening**

	**Genome-wide screening**	**Validation 1**	**Validation 2**
**Cells**	HeLa CD4++ cl1022	HeLa CD4++ cl1022	SupT1
**Format**	Pooled shRNA library	Single shRNA	Single shRNA
**shRNA plasmid**	pSIF H1 SBI (System Biosciences)	pLKO.1 (MISSION® Sigma Aldrich)	pLKO.1 (MISSION® Sigma Aldrich)
**CD4 down-modulation inducer**	LZRS-NA7 Nef-IRES-eGFP	LZRS-NA7 Nef-IRES-eGFP	HIV-1 NL4.3 eGFP

In the second set of experiments, we investigated CD4 down-regulation when Nef alone (thus not the full viral genome) was expressed in SupT1 cells by transduction with a retroviral vector, similar to the approach used in HeLa CD4++ cells for the shRNA genome-wide screening and subsequent validation. Remarkably, only cells knocked down for HRBL, EPS15 and HSP90B1 were found to significantly express higher CD4 surface levels than the non-targeting shRNA control cells. The knock-down of the other gene hits, IDH3G, SNX22, DNM3 caused only a minor, non-significant increase of CD4 surface expression and the knock-down of ATP6AP1 was found to leave the CD4 levels unaffected in this set-up (Figure 3D).

### Effect of the down-regulation of the selected gene products on HIV-1 replication

**Figure 4:**
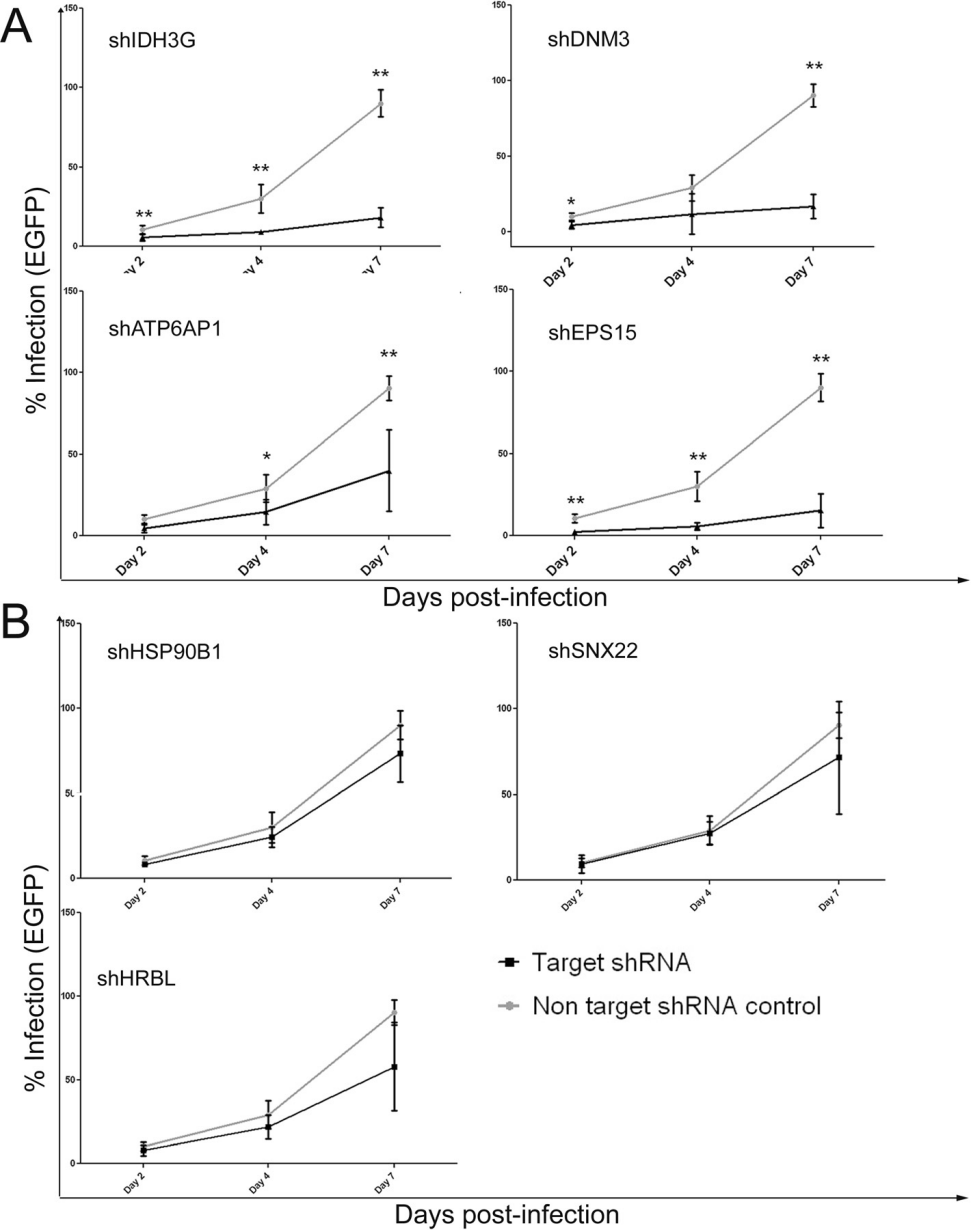
**Knock-down of some genes involved in CD4 down-regulation decreases replication of HIV-1 in SupT1 cells. A**. Knock-down of four genes involved in CD4 down-regulation (EPS15, DNM3, ATP6AP1, IDH3G) decreases HIV-1 replication: graphs show levels of HIV-1 infection at different time points (2, 4 and 7 days post-infection), as measured by the percentage of eGFP expressing cells in the population. The grey lines represent HIV-1 replication in the negative control (cells expressing the non-targeting shRNA), the black line represents cells expressing the target shRNAs. Bar graphs represent the standard deviations for 5 to 6 independent experiments (p values: *p ≤ 0.03, **p ≤0.008). **B**. Knock-down of HRBL, SNX22, HSP90B1 does not affect HIV-1 replication: graph represent levels of HIV-1 infection as described for Figure 4A.

It has been previously shown that Nef-mediated CD4 down-regulation correlates with the ability of the virus to efficiently replicate in CD4+ T lymphocytes [19]. Therefore, we wanted to evaluate whether the rescue of surface CD4 expression in SupT1 cells expressing the selected shRNAs was inhibiting HIV-1 replication. To do so, we evaluated HIV-1 infection levels in SupT1 cells expressing the selected shRNAs two, four and seven days post-infection. By comparing infection levels in these cells with those of cells expressing the non-targeting scrambled shRNA control we found that the knock-down of four out of seven genes (ATP6AP1, IDH3G, DNM3 and EPS15) reduced HIV-1 replication significantly, as shown in Figure 4A, without affecting the viability of the cells (data not shown). Despite increasing CD4 surface expression levels in infected cells, the knock-down of HRBL, HSP90B1 and SNX22 did not have a significant effect on HIV-1 replication (Figure 4B). Therefore, in our experiments, the rescue of CD4 expression levels does not strictly correlate with a decrease in HIV-1 replication.

## Discussion

The importance of HIV-mediated CD4 down-modulation for the viral replication and the disease progression [2-4,20] renders research on this topic of vital importance to better understand the virus and to identify host factors involved in the process. We were especially interested in exploring the cellular pathways that are exploited by the virus to achieve CD4 down-modulation. In order to do so, we screened for host partners involved in HIV-mediated CD4 down-regulation using a genome-wide screening approach in CD4-positive HeLa cells transduced with a retroviral vector encoding the HIV-1 Nef protein as an inducer of CD4 down-modulation [17]. We found several genes known to be involved in CD4 down-regulation by Nef, such as AP-2 [8,9,21] via clathrin-mediated endocytosis [22], illustrating the validity of our genome-wide screening approach. However, others like β-COP were not identified. Such false negatives are inherent to a RNAi screening effort, and can be attributed to the cellular model used, insufficient knock-down or functional compensation by other proteins. Also false positive hits or less relevant host factors might be found, e.g. due to off-target effects of shRNA [23]. To minimize false positives, we chose to validate our findings not only with a different shRNA systems and independent shRNA sequences (individual Mission pLKO.1 vectors opposed to pooled System Biosciences SIF H1 vectors) but also in a different, more relevant cell type and infection model (HIV-1 infected T cells opposed to Nef expressing epithelial cells). Epithelial cell lines have already been successfully used in genome-wide screening studies looking for HIV-1 co-factors or restriction factors [16], however they are not targets for HIV infection. The HeLa CD4++ clone is expressing the receptor artificially by stable transfection and therefore might have a different CD4 expression equilibrium [18]. Furthermore, the HIV-1 Nef protein is only one determinant of the effect of the virus on the CD4 expression profiles. Moreover, as highlighted in the review of Houzet and Jeang [16], a thorough validation of genome-wide screening results is necessary. Finally, validation of screening hits with different shRNA systems and in different cell types contributes to exclude off-target effects. At the end of these stringent validation rounds we were able to identify seven genes which significantly contributed to CD4 down-regulation in HIV-infected SupT1 cells: DNM3, SNX22, HRBL, IDH3G, HSP90B1, EPS15 and ATP6AP1. Additionally, we investigated whether the effects observed in HIV-1 infected SupT1 cells were selectively dependent on Nef. To do so, we compared CD4 levels in cells infected with either a wild type reporter HIV-1 or a HIV-1 carrying a deletion in the *vpu* gene. The fold increase in CD4-expression on cells in which the target genes were knocked-down, relative to the non-targeting shRNA-expressing cells, was comparable for both viruses, suggesting that these hit genes are not important for Vpu-mediated CD4 down-regulation. To investigate Nef specifically, we evaluated CD4 expression levels in cells expressing both the shRNAs targeting the gene hits and Nef. HRBL, EPS15 and HSP90B1 knock-down increased CD4 levels significantly in this experimental set-up, while the knock-down of IDH3G, SNX22 and DNM3 affected CD4 expression levels only in a minor, non-significant manner. The difference between these results and the ones obtained when Nef was expressed in HeLa CD4++ could be due to differences in CD4 endocytosis and trafficking in epithelial cells artificially expressing the CD4 receptor [18]. Compared to infected cells, Nef expression levels might be different, and additional contributions of other HIV proteins like Env to CD4 down-regulation are missing when Nef is expressed alone.

In general the knock-down of these genes does not rescue CD4 levels completely to levels in non-infected cells. The incomplete rescue could be due to partial knock-down of the target genes, as residual expression is still observed via mRNA quantification by qPCR. Furthermore, pathway redundancy might compensate in part for the function of a knocked-down gene candidate involved in CD4 trafficking. Not surprisingly, most of the identified factors are known to be involved in endocytic events at the cell surface or at the level of early endocytic sorting pathways. It has been shown previously that dynamin 2 (DNM2) is required for Nef-mediated CD4 down-regulation [9] but the role of DNM3 has not been investigated before. DNM3, a member of the dynamin protein family, could therefore have an analogous role as a co-factor for HIV-1-mediated CD4 down-regulation. EPS15 is a clathrin adaptor, interacting with AP-2 during vesicle formation. The knock-down of EPS15 is known to strongly reduce Nef- and PMA-dependent CD4 down-modulation [8] and our study is the first to evaluate the role of EPS15 on CD4 down-regulation in productively HIV-1-infected cells. HRBL is a poorly characterized protein, but shares similarity with another Rev binding protein, HRB (HIV Rev Binding protein), which is able to affect endocytosis of transferrin in an EPS15-dependent manner and has been found in the AP-2 interactome [24]. Our findings suggest a role for HRBL in clathrin mediated endocytosis by demonstrating its role in HIV-mediated CD4 down-regulation and propose it to be an interesting candidate for further studies together with its binding partner EPS15. The finding that both EPS15 and HRBL mediate CD4 down-regulation in a Nef-specific manner makes these two genes especially intriguing for further mechanistic investigation. SNX22 is located further in the endocytic pathway, as sorting nexins are involved in early endosomal sorting events. More mechanistic investigation is needed on the role of SNX22 on CD4 down-modulation as its structure and function have been poorly studied so far [25]. ATP6AP1, a V-ATPase, is involved in the acidification of endocytic compartments and could therefore play a role in endocytic sorting or lysosomal degradation of the internalized receptor. It has also been previously shown that V-ATPases interacts with Nef and AP-2 [26]. However, the contribution of ATP6AP1 to HIV-1 mediated down-regulation of CD4 that was observed with one HIV-1 construct (NLENG1-IRES) could not be confirmed in experiments with NL4-3 IRES-EGFP HIV. The role of HSP90B1 and IDH3G in CD4 endocytosis is less clear, as these proteins are not known to be associated with the endocytic and trafficking pathway, although HSP90B1, a chaperone involved in the maturation of proteins in the ER [27], could have an effect on the folding of viral or cellular proteins important for this process. Moreover, HSP90 has been found to promote HIV-1 life cycle in several ways (e.g. by activating the P-TEFb complex necessary for HIV-1 transcription or by enhancing replication in hypothermic conditions) [28]. For IDH3G no role in endocytosis has been shown. Nevertheless, this gene could be very important for HIV-1 replication as it is a key enzyme of the citric acid cycle and necessary for the energetic metabolism of the cell. A transcriptome analysis highlighted the citric acid cycle as one of the pathways enriched in CD4+ and CD8+ cells from HIV-1-infected patients [29]. It has been shown previously that CD4 down-regulation by HIV-1 Nef promotes efficient viral replication [19]. Therefore, we wanted to investigate whether the increased CD4 expression levels in the target shRNA-expressing cells could hamper HIV-1 replication. We found that the knock-down of four genes (DNM3, IDH3G, EPS15 and ATP6AP1) significantly inhibits HIV-1 replication, especially at later time-points (from four to seven days post-infection). We didn’t observe a strict correlation between CD4 down-regulation and HIV-1 replication, as the knock-down of HRBL, SNX22 and HSP90B1 increased CD4 surface expression, but did not affect HIV-1 replication significantly. It could be speculated that the effect on replication by DNM3, IDH3G, EPS15, and ATP6AP1 knock-down are due to functions of these genes independent of HIV-mediated CD4 down-regulation. On the other hand, the knock-down of the three genes with the largest effect on CD4 expression in infected cells, DNM3, IDH3G and EPS15, also had the most detrimental effect on HIV-1 replication. Following this, we hypothesize that the CD4 levels in cells expressing shHRBL, shHSP90B1 and shSNX22 are still sufficiently low to ensure an efficient HIV-1 replication, while the negative effect on HIV-1 replication observed in shATP6AP1 cells could be due to other functions of this protein and be not linked to CD4 expression.

In a study by Daecke J. *et al*. [30], the expression of a dominant negative mutant of EPS15 greatly reduced HIV-1 entry by endocytosis in HeLa cells. Doria *et al*. [31] have also shown that EPS15 could be involved in replication at the level of the Rev export pathway in a synergistic manner with the HIV-1 Rev Binding proteins HRB and HRBL. Considering our results and the data published in literature, it will be interesting to further evaluate the effects of EPS15 and DNM3 on HIV-1 entry and infectivity and the mechanisms involved. Moreover, it will be interesting to evaluate the effects of EPS15 and DNM3 on later replication events (e.g. virion budding).

## Conclusions

In conclusion, we propose a novel genome-wide screening set-up for the study of cellular factors involved in the endocytosis of surface proteins and receptors. This approach could be useful for broader applications, other than the study of HIV-1-mediated CD4 down-modulation. Our results identify seven genes, DNM3, SNX22, HRBL, IDH3G, HSP90B1, EPS15, ATP6AP1 as co-factors for HIV-1-mediated CD4 down-regulation in T cells. The knock-down of EPS15, DNM3, ATP6AP1 and IDH3G also significantly reduces HIV-1 replication in T cells, although more experimental efforts are required to investigate whether the decreased HIV-1 replication can be attributed to changed CD4 expression levels in infected cells or are due to independent functions of these proteins. These findings give insights in the HIV-1-mediated CD4 down-regulation at the level of the plasma membrane and early endosomes and open future research possibilities for the elucidation of the HIV-1-mediated CD4 down-modulation pathway.

## Methods

### Cell cultures

SupT1, 293 T and Phoenix cell lines were cultured as described previously [9], HeLa CD4++ cl1022 (AIDS Research and Reference Reagent Program, National Institutes of Health, Bethesda, MD) cells were cultured in Iscove modified Dulbecco medium (IMDM, Life Technologies, Carlsbad, CA) supplemented with 2 mM L-glutamine 1% (Life Technologies), 10% (vol/vol) heat inactivated fetal calf serum (Hyclone Perbio, Thermo Scientific, Rockford, IL), 100 U/mL penicillin, 100 μg/mL streptomycin (Life Technologies) and geneticin 10 μL/mL (G418 Sulfate, Life Technologies) at 37°C in a humidified atmosphere containing 7% (vol/vol) CO_2_ in air.

### Plasmids and vectors

The GeneNet^TM^ shRNA Lentiviral Library, the control vectors pSIF1-H1 · shLuc-copGFP, pSIF1-H1 · puro-shRNA, pSIF1-H1 · shLuc-puro and the packaging plasmids pFIV-34N and vesicular stomatitis virus envelope pVSV-G were all purchased from System Biosciences (Mountain View, CA). The retroviral vector LZRS-NA7-IRES-eGFP encoding Nef (HIV-1 subtype B NA7 strain) and eGFP was described before [9,16]. The shRNA clones pLKO.1-puro (TRC1 and TRC1.5, 2 to 5 different pLKO.1-shRNA-puro clones with different shRNA sequences per gene depending on availability and validation status) were purchased as bacterial glycerol stocks from Sigma Aldrich (St. Louis, MO). MISSION® Lentiviral Packaging Mix containing lentiviral packaging plasmid and the VSV envelope plasmid were purchased as a pre-made mix from Sigma Aldrich. As control, a pLKO.1 vector encoding a non-targeting scrambled shRNA (a shRNA sequence not targeting any known gene) and puromycin resistance as a marker gene was used (non-targeting-puro shRNA, SHC002, Sigma Aldrich). Plasmid containing cloned proviral DNA of HIV-1 NLENG1-IRES (replication competent HIV NL4-3 virus, characterized by complete HIV genome engineered to express eGFP as part of a bi-cistronic eGFP-IRES-Nef mRNA) was kindly provided by Dr. D.N. Levy, New York University college of Dentistry, New York, NY [32,33]. For the experiments comparing effects on CD4 expression of WT and Vpu-deleted HIV-1, HIV-1 NL4-3 proviral constructs either WT or deleted and engineered to express eGFP as part of a bi-cistronic Nef-IRES-eGFP mRNA were kindly donated by Prof. Dr. Frank Kirchhoff and described in [6].

### Production of retroviral and lentiviral vectors and replication-competent HIV-1 reporter virus

The Phoenix-Amphotropic packaging cell line was transfected with the different retroviral constructs as described previously [9,17]. For lentiviral vector production from pLKO.1 vectors, 293 T cells were seeded 24 h before transfection in 96-wells plates at a density of 13,000 cells per well or in 6-wells plates at a density of 400,000 cells per well. Transfection of the pLKO.1 clones was performed after mixing with MISSION® Lentiviral Packaging Mix using FuGENE® HD (Promega, Madison, WI) following the protocol recommended by the manufacturer. The supernatant was harvested 48 and 72 h after transfection and stored at -80°C until use for transduction of the selected cell lines. Lentivirus production from pSIF1 vectors, was performed using the envelope pVSV-G and packaging pFIV-34 N plasmids (System Biosciences) as described before [9]. Replication-competent HIV-1 virus was produced by transfection (JetPei®, Polyplus, Sélestat, France), according to manufacturer’s instructions, of 293 T cells (seeded 24 hours before transfection at a density of 750,000) with the pNL4-3 proviral construct NLENG1-IRES [32,33]. Viral supernatant was harvested 48 hours after transfection, spun at 900 g for 10 min, to clarify the supernatant from remaining cells and stored at -80°C. The titer of the viral supernatants was measured by quantification of reverse transcriptase activity via real-time PCR as described in [34] and expressed as equivalent p24.

### Monoclonal antibodies, flow cytometry, and cell isolation methods

The monoclonal antibodies used were allophycocyanin (APC)-conjugated human anti-CD4 (Miltenyi Biotec, Leiden, The Netherlands) and phycoerythrin (PE)-conjugated anti-CD4 antibody (Becton Dickinson, Franklin Lakes, NJ). The stained cells were analyzed for CD4 expression on a MACSQuant flow cytometer (Miltenyi Biotec) or FACS Calibur flow cytometer (Becton Dickinson). GFP^+^ HeLa CD4++ cl1022 cells were sorted with FACS Aria cell sorter (Becton Dickinson) after staining with PE-conjugated anti-CD4 antibody. The purity of the isolated cell fractions was analyzed with FACS Calibur (Becton Dickinson). Analysis of flow cytometry data was performed with MACSQuantify^TM^ version 2.5 and Flowing software version 2.5.1.

### Genome-wide shRNA screening

Hela-CD4++ cl1022 cells were transduced either with the pooled shRNA library or the control lentiviral vector H1 · siLuc-copGFP (System Biosciences). Viral titer was adjusted to a multiplicity of infection of 0.3. Efficiently transduced cells were selected with puromycin (0.5 μg/mL, Sigma Aldrich) and transduced with the Nef-expressing retroviral vector. Cells maintaining high CD4 expression despite expression of Nef (Nef(eGFP)^+^CD4^hi^) were sorted by FACS as well as the population expressing Nef and low levels of CD4 (Nef(eGFP)^+^CD4^low^).

To identify the shRNA sequences enriched in Nef(eGFP)^+^CD4^hi^ cells, total RNA was extracted from this cell population and from the Nef(eGFP)^+^CD4^low^ reference population. Reverse transcription was performed starting from 10 μg of total RNA following manufacturer’s instructions (System Biosciences). and shRNA sequences were amplified with a nested PCR using biotinylated primers (System Biosciences) following the manufacturer instructions. These amplified shRNA fragments were then digested with lambda exonuclease to remove the non-biotinylated strand, following manufacturer instructions (System Biosciences) and hybridized on Affymetrix microarray U133 v2-plus (hybridization performed by ServiceXS, Leiden, Netherlands). The procedure was performed for both the Nef(eGFP)^+^CD4^hi^ and Nef(eGFP)^+^CD4^low^ populations, that were compared side by side per screening experiment, and repeated for four independent screening experiments. Data summarization from the raw intensity signals obtained from the microarray hybridization was performed using the software Affymetrix Expression Console v1.1 with MAS5 algorythm [35]. The signal intensities of the Nef(eGFP)^+^CD4^hi^ cells were compared to the signal intensities of Nef(eGFP)^+^CD4^low^ cells, resulting in the selection of the genes over-represented in the Nef(eGFP)^+^CD4^hi^ sorted cells with at least two-fold enrichment compared to the Nef(eGFP)^+^CD4^low^ cells. Among these, only intensities with present (P) or marginal (M) detection call, a measure of the *p*-value implemented in the Mas5 algorythm, were selected. From the four screening experiments we selected a shortlist of 55 genes, to which we added 20 selected from literature. The final list of the 75 selected genes is shown in Additional file 1. We selected genes appearing in multiple screenings or in one screening but with a high Nef(eGFP)^+^CD4^hi^/Nef(eGFP)^+^CD4^low^ ratio, followed by further filtration for biological relevance using the pathway analysis tools DAVID (Database for Annotation, Visualization and Integrated Discovery, [36,37]) and IPA (Ingenuity Pathway Analysis, Ingenuity Systems® [38]).

### Viral gene transfer and HIV-1 infection

For retroviral transduction, HeLa CD4++ cl1022 or SupT1 cells were mixed with retroviral vector supernatant and Dotap (0.2 μg/mL, Roche Diagnostics) and spun for 90 min (950 g, 32°C) as described before [9]. For lentiviral transductions, cells were mixed with the lentiviral vector and spun (30 min, 950 g, 32°C) in the presence of polybrene (8 μg/mL; Sigma-Aldrich). For the cells transduced with the pLKO.1-shRNA-puromycin lentiviruses, puromycin selection was started three days after transduction (1 μg/mL puromycin for SupT1 cells and 0.5 μg/mL for HeLa CD4++ cells, Sigma Aldrich). HIV-1 infection of SupT1 cells was performed in 96-well plates using 50,000 cells/well and HIV-1 virus (20 ng p24/well were used for the NLENG1-IRES NL4-3 virus, 40 ng p24/well for HIV-1 NL4-3 IRES-EGFP WT and 50 ng p24/well for HIV-1 NL4-3 IRES-EGFP dVpu) in a final volume of 200 μL. Cells were spun for 90 min at 950 g at 32°C. NLENG1-IRES NL4-3 was used as such to correspond to a multiplicity of infection (MOI) of 0.01, to have in every independent experiment on average 10% infected cells two days post-infection, 28% infected cells four days post-infection and 90-95% seven days post-infection. The cells infected with HIV-1 NL4-3 IRES-EGFP viruses were analyzed four days post-infection, when the percentage of infected cells was 6% (WT) and 7% (dVpu).

In the experiments requiring both lentiviral (pLKO.1-shRNA-puro) and retroviral (LZRS-NA7-IRES-eGFP) vector transduction or infection with a replication competent HIV-1 virus, the following protocol was applied: cells were transduced with the shRNA lentiviruses and efficiently transduced cells were selected with puromycin as described above. Three days after the start of selection, cells were transduced with the Nef-encoding retrovirus or infected with HIV-1. The degree of CD4 receptor modulation was evaluated by flow cytometry 3 days after transduction or 2, 4 and 7 days after infection for the NLENG1-IRES NL4-3 virus; 2 and 4 days post-infection for HIV-1 NL4-3 IRES-EGFP. The residual CD4 surface expression was calculated as described previously [9].

### Primers

The primers used for the amplification and biotinylation of the shRNA hairpins Fwd-GNF/Rev-GNF and NFwd-Bio/NRev-GNF were included in the shRNA Lentiviral Library kit (System Biosciences) and the PCR/nested PCR were performed following the manufacturer’s instructions.

Primers used for qPCR were: HRBL Fwd (sense) 5’-CCC CCT CGT GTC AAG TC-3’, HRBL Rev (antisense) 5’-CGG CAA ACC TCA TTT CCA CG-3’, SNX22 Fwd (sense) 5’-TGG AAG TTC ACA TCC CGT CG-3’, SNX22 Rev (antisense) 5’-CTC GGA ACA CCA TGT GGC TT-3’, DNM3 Fwd (sense) 5’-TGG CAT GTG ATT CCC AGG AG-3’, DNM3 Rev (antisense) 5’-TCC ATT CTC ATC ATT TTC AGC TAC A-3’, EPS15 Fwd (sense) 5’-TTG CAT TGT TTG CTG GTC TTC T-3’, EPS15 Rev (antisense) 5’-TGA AGA TCC TGA ACC TCA CTT G-3’, ATP6AP1 Fwd (sense) 5’-CGT ACA ACC AGT GGG AG-3’, ATP6AP1 Rev (antisense) 5’-TCA GTG AGA GCC TGG CAA AG-3’; UBC Fwd (sense) 5’- ATTTGGGTCGCGGTTCTTG -3’, UBC Rev (antisense) 5’- TGCCTTGACATTCTCGATGGT-3’, YWHAZ Fwd (sense) 5’-CTTTTGGTACATTGTGGCTTC AA -3’, YWHAZ Rev (antisense) 5’-CCGCCAGGACAAACCAGTAT -3’, ACTB Fwd (sense) 5’-TGACCCAGATCATGTTTGAGA -3’, ACTB Rev (antisense) 5’AGAGGCGTACAGGGATAG GA-3’. These were designed with PrimerBLAST [39] and checked for homo-dimers, hetero-dimers and hairpin formation with IDT OligoAnalyzer [40] (http://eu.idtdna.com/analyzer/applications/oligoanalyzer). All primers were purchased from IDT (Integrated DNA Technologies, Europe Branch, Leuven, Belgium). amplification efficiency was evaluated using a serial cDNA dilution series as standard curve and efficiencies were found to be 90-110% for all primer paires. Primers for IDH3G and HSP90B1 were purchased as PrimePCR Sybr Green assays from Biorad (Nazareth, Belgium).

### Quantitative real-time PCR

Quantitative real-time PCR (qPCR) was used to evaluate the residual expression levels of target genes EPS15, HSP90B1, IDH3G, HRBL, ATP6AP1, DNM3, SNX22 in SupT1 cells expressing the shRNA clones. Reverse transcription was performed with SuperScript® III reverse transcriptase and random primers (Life Technologies), starting from 1 μg of total RNA extracted from the frozen lysate of the transduced cells (RNeasy kit, Qiagen, Venlo, The Netherlands) and treated with DNAse I (Life technologies) following the manufacturer’s instructions. All qPCR reactions were performed using the LightCycler® 480 SYBR Green I Master mix (Roche Applied Science, Vilvoorde, Belgium) in a final volume of 15 μl on the LightCycler® 480 (Roche Applied Science). The primers designed in house with PrimerBlast and purchased from IDT were used at a final concentration of 300 nM. The thermal cycling conditions were 95°C for 10 min, followed by 45 cycles at 95°C for 10 s, 60°C for 30 s and 72°C for 30 s. The relative expression levels of the target genes were calculated using the qBasePLUS software 2.0 (Biogazelle, Zwijnaarde, Belgium) with UBC (ubiquitin C), ACTB (beta-actin) and YWHAZ (tyrosine 3-monooxygenase/tryptophan 5-monooxygenase activation protein) as reference genes, selected with the geNorm algorithm based on their expression stabilities [41]. The expression of the genes relative to the non-targeting scrambled control were calculated with the qBase software (Biogazelle, Belgium).

### Selected shRNA clones

The shRNA clones targeting the selected gene candidates whose knock-down was found to inhibit HIV-1-mediated CD4 down-regulation and/or HIV-1 replication in SupT1 are (The RNAi Consortium Number (TRCN) and NCBI RefSeq transcript number are indicated): EPS15 [TRCN0000060225; NCBI RefSeq: NM_001981], HRBL [TRCN0000011062; NCBI RefSeq: NM_030662], ATP6AP1 [TRCN0000117834; NCBI RefSeq: NM_178509], SNX22 [TRCN0000006650; NCBI RefSeq: NM_004945], HSP90B1 [TRCN0000029425; NCBI RefSeq: NM_003299], IDH3G [TRCN0000027281; NCBI RefSeq: NM_174869], DNM3 [TRCN0000051404; NCBI RefSeq: NM_015569].

### Statistical analysis

Wilcoxon paired test was performed using GraphPad Prism version 5.00 for Windows (GraphPad Software, San Diego California USA).

## Additional files

Additional file 1:
**Summary of the 75 hits chosen after the genome-wide shRNA screening in HeLa CD4++.** The table shows gene symbols and the origin of the chosen hit (screening or literature). When the hit originates from the screening, also the frequency in four independent screenings and the Nef(eGFP)^+^CD4^hi^/ Nef(eGFP)^+^CD4^low^ ratio is listed. Some screening hits are identified twice in the same array by different probes. Additional file 2:
**Description of the 20 preliminary hits from validation 1.** The table describes the 20 hits obtained from validation 1 in HeLa CD4++ cells expressing Nef with the official gene symbol and name and a brief functional description. Additional file 3:
**mRNA expression after shRNA-mediated knock-down for the genes selected in validation 2.** The bar graph shows residual mRNA expression of the indicated target genes after shRNA-mediated knock-down in SupT1 cells, relative to the levels in SupT1 cells transduced with the non-targeting scrambled shRNA control (grey bar) as determined by qPCR. The error bars represent the standard deviation for two to five independent experiments.

## References

[1] Pitcher C, Honing S, Fingerhut A, Bowers K, Marsh M (1999). Cluster of differentiation antigen 4 (CD4) endocytosis and adaptor complex binding require activation of the CD4 endocytosis signal by serine phosphorylation. Mol Biol Cell.

[2] Landi A, Iannucci V, Van Nuffel A, Meuwissen P, Verhasselt B (2011). One protein to rule them all: modulation of cell surface receptors and molecules by HIV Nef. Curr HIV Res.

[3] Watkins RL, Zou W, Denton PW, Krisko JF, Foster JL, Garcia JV (2013). In vivo analysis of highly conserved Nef activities in HIV-1 replication and pathogenesis. Retrovirology.

[4] Casartelli N, Di Matteo G, Potesta M, Rossi P, Doria M (2003). CD4 and major histocompatibility complex class I downregulation by the human immunodeficiency virus type 1 nef protein in pediatric AIDS progression. J Virol.

[5] Chen BK, Gandhi RT, Baltimore D (1996). CD4 down-modulation during infection of human T cells with human immunodeficiency virus type 1 involves independent activities of vpu, env, and nef. J Virol.

[6] Wildum S, Schindler M, Munch J, Kirchhoff F (2006). Contribution of Vpu, Env, and Nef to CD4 down-modulation and resistance of human immunodeficiency virus type 1-infected T cells to superinfection. J Virol.

[7] Stevenson M, Meier C, Mann AM, Chapman N, Wasiak A (1988). Envelope glycoprotein of HIV induces interference and cytolysis resistance in CD4+ cells: mechanism for persistence in AIDS. Cell.

[8] Jin Y-J, Yi Cai C, Zhang X, Hirst JA, Burakoff SJ (2005). HIV Nef-mediated CD4 down regulation is adaptor protein complex 2 dependent. J Immunol.

[9] Stove V, Van de Walle I, Naessens E, Coene E, Stove C, Plum J, Verhasselt B (2005). Human immunodeficiency virus Nef induces rapid internalization of the T-cell coreceptor CD8alphabeta. J Virol.

[10] Schaefer MR, Wonderlich ER, Roeth JF, Leonard JA, Collins KL (2008). HIV-1 Nef targets MHC-I and CD4 for degradation via a final common beta-COP-dependent pathway in T cells. PLoS Pathog.

[11] daSilva LL, Sougrat R, Burgos PV, Janvier K, Mattera R, Bonifacino JS (2009). Human immunodeficiency virus type 1 Nef protein targets CD4 to the multivesicular body pathway. J Virol.

[12] Binette J, Dube M, Mercier J, Halawani D, Latterich M, Cohen EA (2007). Requirements for the selective degradation of CD4 receptor molecules by the human immunodeficiency virus type 1 Vpu protein in the endoplasmic reticulum. Retrovirology.

[13] Magadan JG, Perez-Victoria FJ, Sougrat R, Ye Y, Strebel K, Bonifacino JS (2010). Multilayered mechanism of CD4 downregulation by HIV-1 Vpu involving distinct ER retention and ERAD targeting steps. PLoS Pathog.

[14] Dube M, Bego MG, Paquay C, Cohen EA (2010). Modulation of HIV-1 host interaction: role of the Vpu accessory protein. Retrovirology.

[15] Willey RL, Maldarelli F, Martin MA, Strebel K (1992). Human immunodeficiency virus type 1 Vpu protein induces rapid degradation of CD4. J Virol.

[16] Houzet L, Jeang KT (2011). Genome-wide screening using RNA interference to study host factors in viral replication and pathogenesis. Exp Biol Med.

[17] Stove V, Naessens E, Stove C, Swigut T, Plum J, Verhasselt B (2003). Signaling but not trafficking function of HIV-1 protein Nef is essential for Nef-induced defects in human intrathymic T-cell development. Blood.

[18] Pelchen-Matthews A, Armes JE, Griffiths G, Marsh M (1991). Differential endocytosis of CD4 in lymphocytic and nonlymphocytic cells. J Exp Med.

[19] Lundquist CA, Tobiume M, Zhou J, Unutmaz D, Aiken C (2002). Nef-mediated downregulation of CD4 enhances human immunodeficiency virus type 1 replication in primary T lymphocytes. J Virol.

[20] Mann JK, Byakwaga H, Kuang XT, Le AQ, Brumme CJ, Mwimanzi P, Omarjee S, Martin E, Lee GQ, Baraki B, Danroth R, McCloskey R, Muzoora C, Bangsberg DR, Hunt PW, Goulder PJ, Walker BD, Harrigan PR, Martin JN, Ndung'u T, Brockman MA, Brumme ZL (2013). Ability of HIV-1 Nef to downregulate CD4 and HLA class I differs among viral subtypes. Retrovirology.

[21] Ren X, Park SY, Bonifacino JS, Hurley JH (2014). How HIV-1 Nef hijacks the AP-2 clathrin adaptor to downregulate CD4. eLife.

[22] Foti M, Mangasarian A, Piguet V, Lew DP, Krause KH, Trono D, Carpentier JL (1997). Nef mediated clathrin-coated pit formation. J Cell Biol.

[23] Hao L, He Q, Wang Z, Craven M, Newton MA, Ahlquist P (2013). Limited agreement of independent RNAi screens for virus-required host genes owes more to false-negative than false positive factors. PLoS Comp Biol.

[24] Chaineau M, Danglot L, Proux-Gillardeaux V, Galli T (2008). Role of HRB in clathrin-dependent endocytosis. J Biol Chem.

[25] Cullen PJ (2008). Endosomal sorting and signalling: an emerging role for sorting nexins. Nat Rev.

[26] Geyer M, Yu H, Mandic R, Linnemann T, Zheng YH, Fackler OT, Peterlin BM (2002). Subunit H of the V-ATPase binds to the medium chain of adaptor protein complex 2 and connects Nef to the endocytic machinery. J Biol Chem.

[27] Yang Y, Li Z (2005). Roles of heat shock protein gp96 in the ER quality control: redundant or unique function?. Mol Cells.

[28] Low JS, Fassati A (2014). HSP90: a chaperone for HIV-1. Parasitology.

[29] Wu JQ, Dwyer DE, Dyer WB, Yang YH, Wang B, Saksena NK (2011). Genome-wide analysis of primary CD4+ and CD8+ T cell transcriptomes shows evidence for a network of enriched pathways associated with HIV disease. Retrovirology.

[30] Daecke J, Fackler OT, Dittmar MT, Krausslich H (2005). Involvement of clathrin-mediated endocytosis in human immunodeficiency virus type 1 entry. J Virol.

[31] Doria M, Salcini AE, Colombo E, Parslow TG, Pelicci PG, Di Fiore PP (1999). The eps15 homology (EH) domain-based interaction between eps15 and hrb connects the molecular machinery of endocytosis to that of nucleocytosolic transport. J Cell Biol.

[32] Kutsch O, Benveniste EN, Shaw GM, Levy DN (2002). Direct and quantitative single-cell analysis of human immunodeficiency virus type 1 reactivation from latency. J Virol.

[33] Levy DN, Aldrovandi GM, Kutsch O, Shaw GM (2004). Dynamics of HIV-1 recombination in its natural target cells. Proc Natl Acad Sci U S A.

[34] Vermeire J, Naessens E, Vanderstraeten H, Landi A, Iannucci V, Van Nuffel A, Taghon T, Pizzato M, Verhasselt B (2012). Quantification of reverse transcriptase activity by real-time PCR as a fast and accurate method for titration of HIV, lenti- and retroviral vectors. PLoS One.

[35] Pepper SD, Saunders EK, Edwards LE, Wilson CL, Miller CJ (2007). The utility of MAS5 expression summary and detection call algorithms. BMC Bioinformatics.

[36] Huang DW, Sherman BT, Lempicki RA (2009). Systematic and integrative analysis of large gene lists using DAVID Bioinformatics Resources. Nat Protoc.

[37] Huang DW, Sherman BT, Lempicki RA (2009). Bioinformatics enrichment tools: paths toward the comprehensive functional analysis of large gene lists. Nucleic Acids Res.

[38] Ingenuity Pathway Analysis (IPA) (Ingenuity® Systems, www.ingenuity.com).

[39] PrimerBLAST, http://www.ncbi.nlm.nih.gov/tools/primer-blast/.

[40] IDT OligoAnalyzer (http://eu.idtdna.com/analyzer/applications/oligoanalyzer, Integrated DNA Technologies, Europe Branch, Leuven, Belgium).

[41] Vandesompele J, De Preter K, Pattyn F, Poppe B, Van Roy N, De Paepe A, Speleman F (2002). Accurate normalization of real-time quantitative RT-PCR data by geometric averaging of multiple internal control genes. Genome Biol.

