# Supplementary figures and images for: Genome-wide shRNA screening identifies host factors involved in early endocytic events for HIV-1-induced CD4 down-regulation
Source: Retrovirology. 2014 Dec 13;11:118. doi: 10.1186/s12977-014-0118-4 (PMC4269872; doi:10.1186/s12977-014-0118-4)

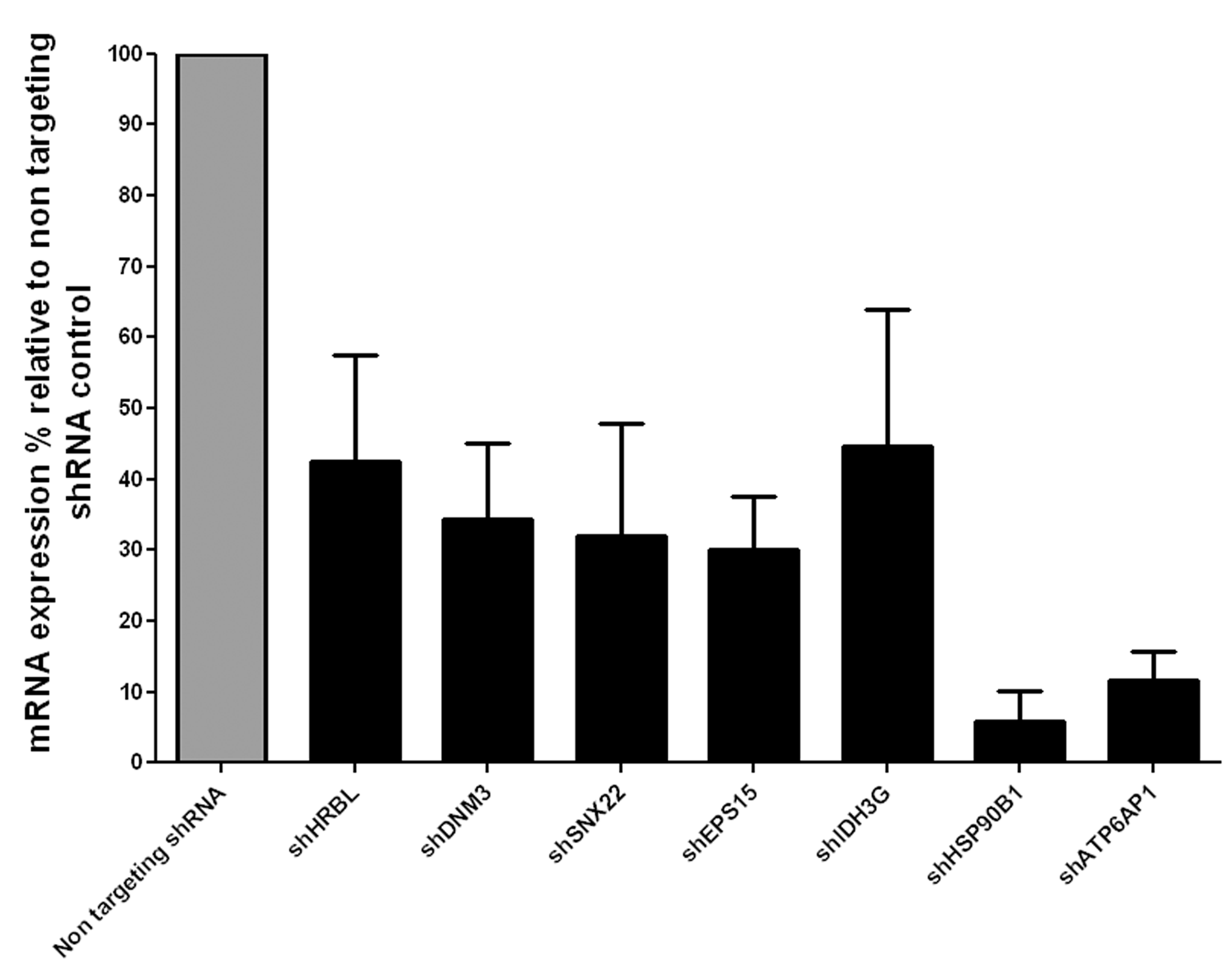

Supplement: Additional file 3: — mRNA expression after shRNA-mediated knock-down for the genes selected in validation 2. The bar graph shows residual mRNA expression of the indicated target genes after shRNA-mediated knock-down in SupT1 cells, relative to the levels in SupT1 cells transduced with the non-targeting scrambled shRNA control (grey bar) as determined by qPCR. The error bars represent the standard deviation for two to five independent experiments. [file 12977_2014_118_MOESM3_ESM.tiff]
